# Association of right atrial structure with incident atrial fibrillation: a longitudinal cohort cardiovascular magnetic resonance study from the Multi-Ethnic Study of Atherosclerosis (MESA)

**DOI:** 10.1186/s12968-020-00631-1

**Published:** 2020-05-21

**Authors:** Eric Xie, Ricky Yu, Bharath Ambale-Venkatesh, Hooman Bakhshi, Susan R. Heckbert, Elsayed Z. Soliman, David A. Bluemke, Steven M. Kawut, Colin O. Wu, Saman Nazarian, João A. C. Lima

**Affiliations:** 1 Cardiology Division, Department of Medicine, Johns Hopkins Hospital, Blalock 524D, 600 North Wolfe Street, Baltimore, MD 21287 USA; 2 Heart Service, Department of Medicine, UCLA School of Medicine, Los Angeles, CA USA; 3 Department of Radiology, Johns Hopkins Hospital, Baltimore, MD USA; 4 Department of Epidemiology, School of Public Health, University of Washington, Seattle, WA USA; 5 Department of Epidemiology and Prevention, Epidemiological Cardiology Research Center, Wake Forest School of Medicine, Winston-Salem, NC USA; 6 Department of Medicine, Section of Cardiology, Wake Forest School of Medicine, Winston-Salem, NC USA; 7 Radiology and Imaging Sciences, National Institutes of Health, Bethesda, MD USA; 8 Department of Medicine, Perelman School of Medicine at the University of Pennsylvania, Philadelphia, PA USA; 9 Office of Biostatistics Research, National Heart, Lung and Blood Institute, National Institutes of Health, Bethesda, MD USA

**Keywords:** Cardiovascular magnetic resonance, Feature tracking, Atrial volume, Strain, Atrial fibrillation

## Abstract

**Background:**

While studies of the left atrium (LA) have demonstrated associations between volumes and emptying fraction with atrial fibrillation (AF), the contribution of right atrial (RA) abnormalities to incident AF remains poorly understood.

**Objectives:**

Assess the association between RA structure and function with incident AF using feature-tracking cardiovascular magnetic resonance (CMR).

**Methods:**

This is a prospective cohort study of all participants in the Multi-Ethnic Study of Atherosclerosis with baseline CMR, sinus rhythm, and free of clinical cardiovascular disease at study initiation. RA volume, strain, and emptying fraction in participants with incident AF (*n* = 368) were compared against AF-free (*n* = 2779). Cox proportional-hazards models assessed association between variables.

**Results:**

Participants were aged 60 ± 10 yrs., 55% female, and followed an average 11.2 years. Individuals developing AF had higher baseline RA maximum volume index (mean ± standard deviation [SD]: 24 ± 9 vs 22 ± 8 mL/m^2^, *p* = 0.002) and minimum volume index (13 ± 7 vs 12 ± 6 mL/m^2^, *p* < 0.001), and lower baseline RA emptying fraction (45 ± 15% vs 47 ± 15%, *p* = 0.02), peak global strain (34 ± 17% vs 36 ± 19%, *p* < 0.001), and peak free-wall strain (40 ± 23% vs 42 ± 26%, *p* = 0.049) compared with the AF-free population. After adjusting for traditional cardiovascular risk factors and LA volume and function, we found RA maximum volume index (hazards ratio [HR]: 1.13 per SD, *p* = 0.041) and minimum volume index (HR: 1.12 per SD, *p* = 0.037) were independently associated with incident AF.

**Conclusions:**

In a large multiethnic population, higher RA volume indices were independently associated with incident AF after adjustment for conventional cardiovascular risk factors and LA parameters. It is unclear if this predictive value persists when additional adjustment is made for ventricular parameters.

## Introduction

Atrial fibrillation (AF) is the most common cardiac arrhythmia in the United States, with a lifetime risk of 1-in-4 for persons over 40 years of age and associated increases in morbidity and mortality [1, 2]. Much of the literature on the influence of cardiac structural abnormalities on AF in patients with subclinical heart disease has focused on the left heart [3–6]. Left atrial (LA) functional and structural parameters have been found to be associated with the risk of AF development and are suspected to be either causative or intermediaries in the pathophysiology of AF. Right ventricle (RV) morphology, emptying fraction, and mass, have also been shown to be associated with risk of AF, possibly as biomarkers of pulmonary dysfunction and hypertension [7]. In contrast, the impact of right atrial (RA) structural abnormalities on risk of AF in patients with subclinical heart disease has been less studied.

In patients with subclinical heart disease, risk factors, including venous thromboembolism (VTE) and pulmonary embolism (PE) [8], obstructive sleep apnea (OSA) [9], and chronic obstructive pulmonary disease (COPD) [10, 11], are thought to mediate AF through their effects on the right heart. Recently, it has been shown that anatomical and electrical abnormalities of the RA can impact ablation outcomes. Most patients have multiple etiologies contributing to their AF, sometimes in both atria [12, 13]. Though ablation of LA mechanisms is often sufficient for rhythm control in paroxysmal AF, a significant minority of paroxysmal AF cases require addressing RA mechanisms as well and effective strategies for ablation of persistent AF remain unresolved [14]. Among patients with persistent AF who underwent first-time catheter ablation with pulmonary vein isolation and LA ablation, enlarged RA volume on 3-D computed tomography (CT) was associated with failure to achieve AF termination [15].

Cardiovascular magnetic resonance (CMR) is well-established for simultaneously assessing LA volume and strain as it potentially provides greater spatial resolution and excellent demarcation of endocardial and epicardial borders [16, 17]. Feature-tracking in CMR is an established method for tracking and assessing wall motion [18–20]. Analyses in the Multi-Ethnic Study of Atherosclerosis (MESA) of CMR-measured parameters have previously demonstrated association of LA strain and function with AF [21, 22]. However, CMR feature-tracking has not been applied in the investigation of the potential contribution of RA structural and functional abnormalities to incident atrial fibrillation in populations.

In this study, we sought to evaluate the role of altered RA structure and function, including peak global and free-wall strain, volumes, and emptying fraction (EF), to the development of AF in a large multiethnic population free of clinically-recognized cardiovascular disease at baseline. We hypothesized that increased RA size and reduced RA function (as assessed by EF and strain) are associated with greater risk of incident AF.

## Methods

### Subjects

**Fig. 1:**
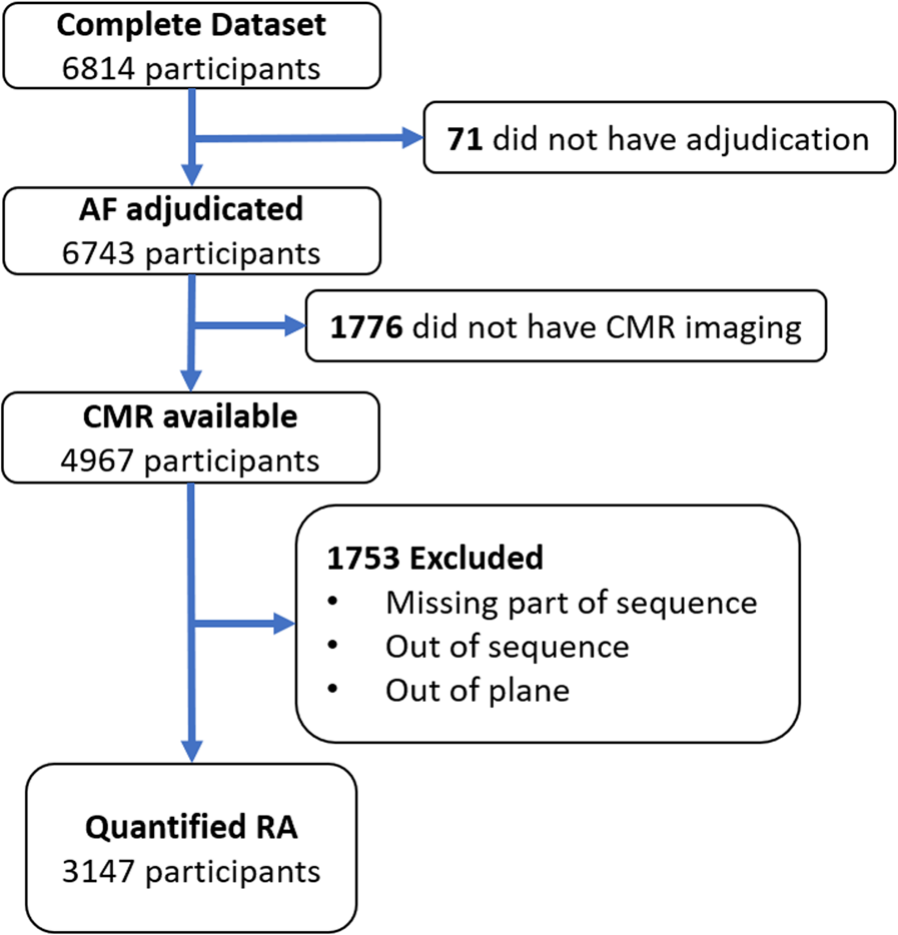
Study flowchart. Flowchart demonstrates number of participants included and excluded resulting in final study population

MESA is a population-based cohort of participants free of clinically-evident cardiovascular disease at baseline. The MESA protocol has previously been described in detail [23]. In brief, 6814 participants were recruited from six US communities between July 2000 and Aug 2002, aged between 45 and 84 years from four different, self-reported racial-ethnic backgrounds. The protocol was approved by the institutional review board at each study site and all participants provided written informed consent. Approximately every 9 months, participants were contacted to inquire as to cardiovascular diagnoses and events, hospital admissions, and mortality. Medical records and information were successfully obtained on an estimated 98% of reported hospitalized cardiovascular events and 95% of reported outpatient cardiovascular diagnostic encounters. Study inclusion and exclusion are shown in the flowchart (Fig. 1).

### Identification of AF cases

Incident cases of AF were identified through December 2014. Cases were identified through MESA hospitalization surveillance, study electrocardiograms (ECGs), and for participants enrolled in fee-for-service Medicare, from inpatient and outpatient Medicare claims data. We excluded individuals who developed AF after coronary artery bypass or valve surgery or those who were found to have an AF claim occurring before their baseline MESA examination.

### CMR protocol

The CMR protocol has previously been described in detail and was performed at time of enrollment [24]. In brief, baseline images were acquired with 1.5 T scanners (Signa, General Electric Healthcare, Waukesha, Wisconsin, USA; Symphony or Sonata, Siemens Healthineers, Erlangen, Germany). Long-axis cine images were obtained in the 4-chamber view using ECG-gated breath hold fast gradient-echo pulse sequence. This view was acquired as one slice of thickness 6 mm and the matrix size was 256 × 160. Temporal resolution varied, averaging 50 ms.

### Image analysis

**Fig. 2:**
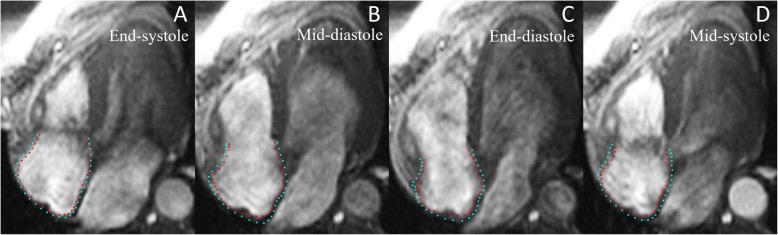
Cardiovascular magnetic resonance (CMR) feature-tracking of right atrial (RA) wall motion. Multimodality tissue-tracking software was used to track CMR images in the 4-chamber view to track right atrial wall motion throughout systole and diastole. Phases of the cardiac cycle are presented in temporal order: end-systole (**a**), mid-diastole (**b**), end-diastole (**c**), mid-systole (**d**). Endocardial and epicardial borders shown in red and cyan, respectively

**Fig. 3:**
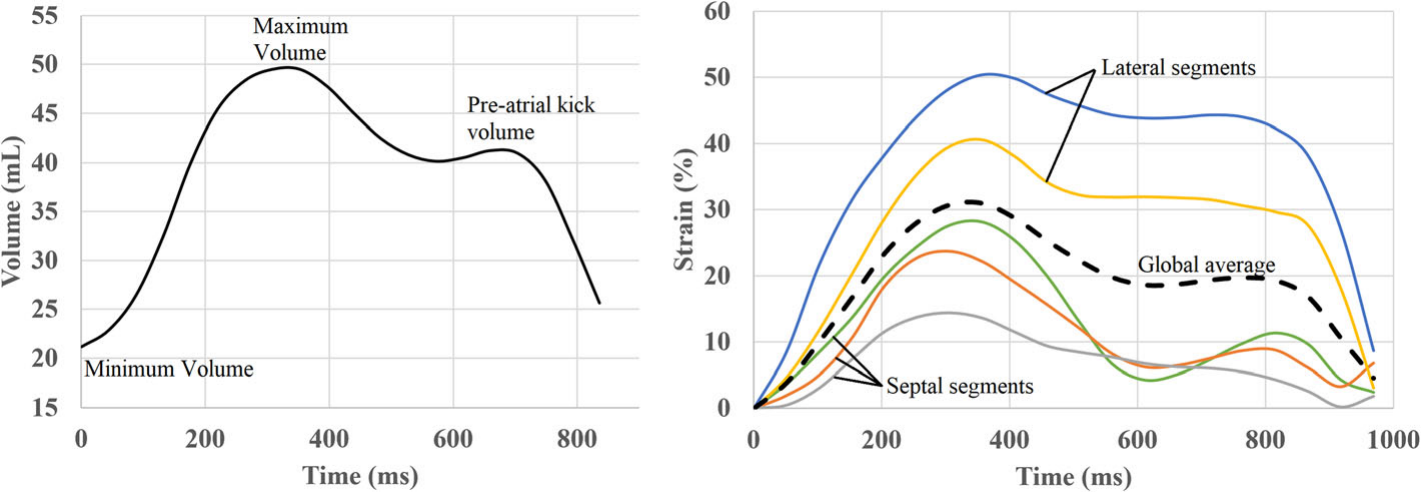
Volume and strain curves derived from feature tracking. Examples of phasic volume/time (left) and strain/time (right) curves demonstrating right atrial (RA) volume and global strain respectively, in black. Also demonstrated are strain curves of different segments in color, on right. The blue and yellow lines correspond with the lateral wall, green line with the basal wall, and orange and grey lines with the septal wall. Volume measurements on left. Strain measurements were taken at maximum (solid black) and again after excluding the septal segments, on **right**

RA function was assessed using multi-modality tissue tracking software (MTT; Toshiba,

Tokyo, Japan), which has been previously described at length [25]. This software has been extensively validated in the LA with excellent reproducibility [26]. We included participants from the MESA cohort who had adequate views of the RA for assessment. Four-chamber cine CMR images were used for analysis. The study was conducted by a single experienced operator blinded to the cardiovascular disease (CVD) outcomes of participants. Endocardial borders were manually traced along the RA at the end-systolic frame and the epicardial border was automatically defined by software at a fixed distance of 3 mm from the manually-traced endocardial border. The borders were then propagated across frames of the cardiac cycle via multimodality tissue-tracking software (MTT) and manually verified for quality control (Fig. 2). The software then generated volume vs time curves from which maximum, minimum, and pre-atrial kick RA volumes were extracted (Fig. 3). The RA volume metrics included in this study are as follows:

- Maximum RA volume: RA volume at end-systole before tricuspid valve opening
- Minimum RA volume: RA volume at end-diastole before tricuspid valve closure

RA EF was calculated based on RA volumes at different time points in the cardiac cycle, as follows:

$$ total\  RA\  EF:\frac{RAV_{max}-{RAV}_{min}}{RAV_{max}} $$

Strain measurement in 4-chamber cine images was automated within the software with segmentation of the borders into free-wall and septal distributions. Strain is computed as an average across the entire manually-defined cardiac contour. The zero-strain reference was set at RA end-diastole. Peak global strain refers to the maximum calculated strain during the cardiac cycle within the RA, which in these baseline measurements of a CVD-free population reflect RA reservoir strain. Free-wall strain refers to the RA lateral wall and RA roof, excluding the septal wall. Strain curves were computed for both peak global and free-wall longitudinal segments during the cardiac cycle and visually assessed for quality.

Study quality was defined categorically from 1 to 4. A single operator was responsible for assigning quality across all studies. Definitions are as follows:

1. Poor quality: Images were missing frames, out of plane, or out of sequence. Tracking was not possible in the MTT software as it was unable to propagate the manual contour. These were excluded from analysis.
2. Fair quality: Images and tracking were acceptable but the cardiac cycle was incompletely captured or contrast was poor causing inaccurate tracking in part of the cardiac cycle
3. Good quality: Images and tracking were without the above issues but required minor operator adjustments
4. Excellent quality: Images and tracking required no operator adjustment

### Covariates

Standardized questionnaires were used at baseline to collect information from participants about demographics and cardiac risk factors as previously described [21]. We screened the following covariates for our analysis: age, sex, race/ethnicity, height, weight, body mass index (BMI), body surface area (BSA), cigarette smoking status, alcohol consumption, diastolic blood pressure (BP), systolic BP, fasting glucose, diabetes, use of hypertension medication, total cholesterol, high density lipoprotein (HDL) cholesterol. Baseline adjudication of COPD, deep venous thrombosus (DVT), PE, or OSA were not available in MESA.

Our multivariate Cox models adjusted for these covariates as follows. In model 1, we included demographic characteristics (age, race, sex) and traditional cardiovascular risk factors (BMI, smoking status, HDL, lipid-lowering medication, systolic BP, hypertension medication, fasting glucose, alcohol use). RA volume index (RAVI_max_) and RAVI_min_ remained significantly associated with incident AF. RA EF, peak global and free-wall strain were not significant after this adjustment. In model 2, we made additional adjustments for LA maximum volume index, peak LA strain, and LA EF as obtained from previous studies [27].

Additionally, we adjusted for LA, left ventricular (LV), and RV variables as obtained from and described in previous studies in MESA [5, 7, 21]. In brief, the LA variables most closely associated with incident AF were: elevated LA maximum volume index (adjusted HR 1.38, *p* = 0.04), passive LA EF (adjusted HR 0.55, *p* < 0.001), and peak LA strain (adjusted HR = 0.68, *p* = 0.031). In the LV, LV mass was the strongest association with AF (adjusted HR = 1.45, *p* < 0.001). For the RV, EF (adjusted HR 1.15, *p* = 0.04) and RV mass (adjusted HR = 1.16, *p* = 0.07) were the most significant associations for AF.

### Statistical analysis

Statistical analysis was performed with Stata software (Stata Corp, College Station, Texas, USA). Continuous variables are denoted here as mean ± standard deviation while categorical variables are denoted as numbers and percentages. Baseline participant characteristics and RA metrics were evaluated using the Student’s T-test for continuous variables and Chi-square test for categorical variables. ANOVA with Tukey’s post-hoc test was used to assess for variation in RA metrics by image quality. Cox proportional hazard models were used to assess the association of RA structure and function parameters with time to incident AF, with death, end of follow-up, and withdrawals treated as censored. Cox models were adjusted for significant predictors of AF selected from among the covariates listed above. Propensity score matching was used for validation of our results. We modeled the odds of AF for participants with a below-median RAVI_max_ compared with above-median RAVI_max_, with matching on demographic and traditional risk factor covariates. *P*-value < 0.05 was considered significant. GraphPad Prism (GraphPad Software, La Jolla, California, USA) was used to create the additional figure.

We evaluated the reproducibility of both RA volume and function metrics in MTT. Interobserver and intraobserver variability was assessed with 20 randomly-chosen cases. RA analysis was independently performed by two operators (E.X. and E.C., a cardiac technologist with 10 years of CMR experience) while blinded to AF incidence and other clinical parameters. Intraclass correlation coefficient analysis was performed to evaluate variability.

## Results

All available CMRs from participants without AF at baseline were screened for inclusion (4967 participants). RA parameters were able to be obtained from CMRs of 3214 participants with fair or better quality. Of these, 3147 participants had AF data and comprised the study population. Of all included studies, 16% were of fair quality, 80% were of good quality, and 4% were of excellent quality. Participants were followed for an average of 11.2 ± 3.7 years. There were 368 participants who developed AF with a mean time to incident AF of 7.4 ± 3.7 years. The proportion developing AF in our participants is not significantly different than the proportion developing AF in all participants with CMR (11.7% vs 12.6%, *p* = 0.25), nor was the proportion of developing AF among participants with CMR different from that in the whole MESA cohort (*p* = 0.37).

The baseline characteristics of participants who did and did not develop AF are summarized in Table 1. Generally, patients who developed AF were older (69 vs 61 years). Caucasian patients developed AF at a higher rate than non-Caucasian patients (15% vs 9.6%). Additionally, higher systolic BP, higher fasting glucose, hypertension medication use, lipid-lowering medications use, and smoking history were associated with incident AF.

**Table 1:** Baseline characteristics of patients who did and did not develop atrial fibrillation during follow-up

	Developed AF *N* = 368 (11.7%)	No AF *N* = 2779 (88.3%)	*P* value
Age (years), mean ± SD	69.2 ± 8.0	61 ± 10	< 0.001
Males, n(%)	188 (51%)	1231 (44%)	0.01
Race, n(%)			< 0.001
Caucasian	183 (50%)	1032 (37%)	
Hispanic	57 (16%)	603 (22%)	
African American	85 (23%)	784 (28%)	
Chinese	43 (11%)	361 (13%)	
Smoker (past or current), n(%)	204 (55%)	1327 (48%)	0.004
BMI (kg/m^2^), mean ± SD	27.9 ± 5	27.9 ± 5.6	0.85
Systolic blood pressure (mmHg), mean ± SD	133 ± 23	123 ± 20	< 0.001
Diastolic blood pressure (mmHg), mean ± SD	72 ± 10	72 ± 10	0.89
Taking blood pressure medication, n(%)	180 (49%)	880 (32%)	< 0.001
Total cholesterol (mg/dl), mean ± SD	195 ± 35	195 ± 36	0.78
HDL (mg/dl), mean ± SD	52.4 ± 16.2	51.4 ± 14.9	0.24
Taking lipid lowering medications, n(%)	81 (22.1%)	414 (14.9%)	< 0.001
Fasting glucose (mg/dL), mean ± SD	102 ± 37	97 ± 31	0.009
Diabetes, n(%)			0.054
Impaired fasting glucose	50 (14%)	343 (12%)	
Diabetes	64 (17%)	365 (13%)	
Alcohol Use			0.34
Current	198 (58%)	1574 (57%)	
Former	94 (26%)	615 (22%)	

Values are mean ± SD or %

*AF* atrial fibrillation, *SD* Standard deviation, *BMI* Body mass index, *HDL* High density lipoprotein

### RA structure and function

RA volume was indexed by BSA yielding maximum volume indexed (RAVI_max_) and minimum volume indexed (RAVI_min_). RA structure and function in patients who did and did not develop AF are summarized in Table 2. The baseline RAVI_max_ was 6% higher and RAVI_min_ is 9% higher in patients with incident AF (23.7 ± 9.2 vs 22.4 ± 8.0 mL/m^2^, *p* = 0.002, 13.2 ± 6.7 vs 12.1 ± 5.8 mL/m^2^, *p* < 0.001, respectively). The RA EF was 4% lower in AF cases (*p* = 0.02). In parallel, baseline RA peak global strain and free-wall strain were 6% lower in AF cases (*p* < 0.001 and *p* = 0.049 respectively).

**Table 2:** Baseline right atrial (RA) parameters overall, in AF cases and those who did not develop AF

RA Parameter (mean ± SD)	Developed AF (*n* = 368)	No AF (*n* = 2779)	*P*-value
RA maximum volume indexed (mL/m^2^)	23.7 ± 9.2	22.4 ± 8.0	0.002
RA minimum volume indexed (mL/m^2^)	13.2 ± 6.7	12.1 ± 5.8	< 0.001
RA total EF %	44.8 ± 15.2	46.6 ± 15.1	0.02
RA global strain %	34.0 ± 17.3	36.2 ± 19.2	< 0.001
RA free-wall strain %	39.9 ± 22.7	42.3 ± 25.9	0.049

Volume indexed to ml/m^2^ BSA

*EF* emptying fraction, *SD* standard deviation, *HR* hazards ratio, *CI* confidence intervals

### Association of RA parameters and incident AF

Multivariable Cox regression models were used to assess the association of baseline RA structure and function metrics with the development of AF (Table 3). Results are presented as HR per SD. Unadjusted, all RA measures were significant.

**Table 3:** Association of right atrial (RA) structure and function with incident AF

RA Parameter	Unadjusted		Model 1		Model 2	
	HR (95% CI)	*P*-value	HR (95% CI)	*P*-value	HR (95% CI)	*P*-value
RA maximum volume indexed	1.16 (1.05,1.28)	0.003	1.24 (1.12,1.36)	< 0.001	1.13 (1.00,1.27)	0.041
RA minimum volume indexed	1.18 (1.08,1.3)	0.001	1.19 (1.08,1.31)	< 0.001	1.12 (1.01, 1.25)	0.037
RA total EF %	0.89 (0.8,0.99)	0.03	0.95 (0.85,1.06)	0.34	0.94 (0.83, 1.07)	0.36
RA global strain %	0.88 (0.79,0.98)	0.02	1.06 (0.95,1.18)	0.32	1.05 (0.92. 1.19)	0.45
RA free-wall strain %	0.9 (0.81,1.0)	0.06	1.02 (0.92,1.14)	0.63	1.01 (0.89, 1.15)	0.82

Hazard ratios are presented per SD change in measurements, with SD noted in Table 2

Model 1 was adjusted for demographics and traditional risk factors: age, race, gender, BMI, smoking status, HDL, lipid-lowering medication, systolic blood pressure, hypertension medication, fasting glucose, alcohol use

Model 2 was additionally adjusted for LA maximum volume index, LA passive emptying fraction, LA peak longitudinal strain

*CI* confidence interval, *LA* left atrium; other abbreviations in Tables 1 and 2

Larger RA structure as assessed by higher RAVI_max_ and RAVI_min_ remained positively associated with incident AF (HR per SD 1.13 and 1.12, respectively). Comparatively, the effects of LA metrics in the model were: LA maximum volume index (HR per SD: 1.46), LA passive EF (HR per SD: 0.79), LA peak strain (HR per SD: 0.82). Propensity score results yielded a similar direction. The top quantile of RAVI_max_ was associated with a 3.5% higher incident AF (*p* = 0.007) at 1:1 matching for AF and non-AF participants. In adjusted quantile analyses of RAVI_max_, there was a notable graded increase in risk with increasing quantiles. Compared to the middle quintile, the 4th and 5th quintiles had, respectively: HR 1.4, *p* = 0.04 and HR = 1.7, *p* = 0.002. In contrast, risk did not appear to be mitigated with lower RA volumes and the lowest quintiles were not significantly different than the middle quintile.

Additionally, correlation between AF risk factors (including those in CHA_2_DS_2_-VASc) and RA volumes is shown (Additional file 1).

We additionally created models adjusting for demographics, traditional risk factors, and RV variables previously identified as being associated with incident AF [7] (Additional file 2). RAVI_max_ was significant in fully adjusted models including RV EF and mass (HR per SD: *1.15, p = 0.01*). RAVI_min_ was not significant after adjustment (HR per SD: *1.11, p = 0.09*). Further adjustment for LV mass had no effect on the model. Neither RAVI_max_ nor RAVI_min_ were significant after adjustment for demographics, traditional risk factors, and both LA and RV variables within the same model.

### Measurement reproducibility and variation of strain and function with image quality

Reproducibility of RA was evaluated in 20 randomly selected subjects. Intraclass correlation coefficients (ICC) for intraobserver reproducibility were 0.93 for RAV_max_, 0.89 for RAV_min_, 0.91 for global strain, and 0.89 for free-wall strain. ICC for interobserver reproducibility were 0.93 for RAV_max_, 0.85 for RAV_min_, 0.85 for global strain, and 0.82 for free-wall strain.

The sub-population of MESA who underwent CMR examination, as expected, was healthier than the sub-cohort who did not (Additional file 3). Within the sub-population who underwent CMR and entered the analysis, measured parameters were significantly different at different image/tracking qualities (Additional file 4). To account for this, interaction between image/tracking quality and RA parameters were incorporated into the preliminary assessment of the Cox models as covariates. They were not found to affect the association with AF for any RA measure and there was no evidence for interaction. Therefore, image quality variables were not included in the final models.

## Discussion

In a large multiethnic population of individuals free of clinically-recognized AF and other CVD at baseline, larger RA structure as measured by CMR was associated with development of AF over and above LA morphological and functional abnormalities. Indeed, higher RAVI_max_ and RAVI_min_ at the MESA baseline examination were associated with incident AF independent of traditional CVD risk factors and LA measures.

We report RA volumes that are comparable to those in the literature, though these studies have generally been conducted with 2D or 3D echocardiography [28–31]. Literature on RA strain is limited and has previously been obtained using speckle-tracking echocardiography in healthy populations [32, 33]. In comparison to these studies, we reported lower average RA strain (36% vs 49%) though we note significant differences in our population including much older participants (mean age 60 vs 34 years), as well as differences in methodology (CMR versus echocardiography). Our population also had significantly more medical comorbidities, higher rates of current smoking, and higher BMI that likely contributed to relatively poorer atrial function and lower strain [34].

Several studies have demonstrated that LA structural and functional changes are present in asymptomatic individuals, years before the development of electrical or mechanical heart disease, including AF and heart failure [21, 25, 35]. In contrast, the RA has been poorly studied and generally excluded from clinical criteria and guidelines. Further determination of its contribution to disease will be necessary for a more complete understanding of the risk of AF development.

### RA structure and incident AF

As structural changes in the LA are well-understood to precede the development of AF [6, 36, 37], we had hypothesized that there would be comparable findings in the RA. Our study corroborated our hypothesis, as RA volume was significantly associated with incident AF in all models. We suspect RA volume serves as an intermediate marker between pathologic processes and AF. It has been demonstrated that in pulmonary arterial hypertension and systolic heart failure, RA volume is significantly increased [30, 38]. An enlarged RA at baseline may reflect subclinical fibrosis, pulmonary insult, or other etiologies preceding detectable disease. In particular, the pathophysiology of AF associated with pulmonary disease may be mediated through the RV and in this study, we demonstrate that RA alterations remain as a significant predictor of AF after adjustment for RV variables. Moreover, early RA remodeling may be a reversible process as has been shown of early LA remodeling [39]. Clinical studies and computational models have indicated that re-entrant drivers of AF in both RA and LA may be associated with fibrotic substrate [40, 41]. In turn, small studies have suggested that markers for increased fibrosis in the LA may be correlated with increased volume index, a potential pathophysiologic link which merits further study in the RA [42]. As the natural history of AF is marked by both structural and electrical remodeling becoming increasingly challenging to treat with increasing burden of disease, appropriate therapies are necessary in both atria to address time-critical mechanisms including development of fibrosis [41].

Existing literature on the association of RA volume with AF is limited. We could not find studies in participants free of CVD. A previous study of RA remodeling after ablation of paroxysmal AF suggested that larger volumes were associated with increased risk of recurrence [43]. However, a similar study of RA volume before ablation found no significant association with AF recurrence [44]. In patients with paroxysmal AF on pharmacological control, RA volume was not associated with maintenance of sinus rhythm [45]. In patients with atrial septal defects, RA volume was not associated with development of AF [46]. However, as these studies followed patients for less than a year, it is also possible that RA volume was a less powerful biomarker in the short follow-up timescale.

The absolute differences in baseline RA volume in patients who did and did not develop AF may be underrepresented as the majority of AF originates from sources in the LA [12, 13]. Though our models suggest a contribution of RA volume to AF risk, the magnitude of the unindexed baseline difference in simple comparison tests, on the order of 4 mL, may be challenging to clinically appreciate. Though most patients have multiple mechanisms of AF development, sometimes involving both atria, ablation of LA sources is often sufficient for rhythm control in upwards of 70% of patients [14]. It is thus likely we had a smaller subset of cases of AF arising predominantly from the RA. This is further supported in our multivariate models which suggest that LA parameters have a larger effect size than RA parameters, though both are independent and significant predictors. However, since it is not infrequent to find RA mechanisms for AF among patients who fail LA ablation procedures to treat AF, recognition of these differences may improve medical management and guide procedural intervention in otherwise refractory cases. It is important to note that when RV variables were incorporated into our models alongside RA and LA variables, RA and RV variables lost significance while LA variables remained significant, a manifestation of the collinearity between RA and RV which makes it challenging to claim in our study that the RA is a predictor entirely independent of other chambers, unlike the LA.

### RA function and incident AF

The healthy RA modulates systemic venous return to ventricular filling in three phases: as a reservoir during systole, as a conduit in early diastole, and as a pump in late diastole. Peak global strain occurs during the second phase, when the ventricle is being passively filled, and serves as a marker for RA compliance and function. In this study, we also measured the free-wall strain, which excludes the atrial septum at this phase. Large studies of CMR-derived strain and LA emptying fraction (LA EF) have highlighted their potential role as predictors of AF [21].

As with RA volume, literature on the relationship between RA function and incident AF is limited, the few existing studies having been conducted in symptomatic patients with cardiac disease. RA emptying fraction was not associated with development of AF in patients with a pathological atrial septal defect undergoing repair [46]. In patients with paroxysmal AF, total peak strain was not associated with greater rate of maintenance of sinus rhythm at 1 year [45]. Our results are consistent with these findings as we did not find associations of peak global strain or RA EF with AF after adjustment for risk factors and subclinical CV disease. This is first study assessing RA function as a predictor of AF in a CVD-free multi-ethnic population. Interestingly, groups have investigated LA EF across different phases (total, passive, and active) though report differing results as to associations with AF [21, 47, 48]. This may be another area of potential future study.

### Limitations

Our study has several limitations. A single reader was responsible for post-processing though reproducibility studies were excellent with multiple readers. Use of a single reader reduces variability in research as in this study but does not reflect general clinical usage. Our study had a higher rate of unusable or uninterpretable images than comparable studies featuring LA wall tracking. Examples of poor quality images which were excluded are shown (Additional file 5). We posit that these differences result from less emphasis on RA than LA imaging during the original CMR acquisition process. Though our quantification methods were rigorously validated in the LA, we do not have standalone validation in the RA. As the MTT software used one slice to compute volume, the calculated volumes may slightly underestimate true volumes [49, 50]. As noted in the Results, there were differences in our measurements in association with RA image quality, which we suspect are due to limitations of feature tracking in noise and artifact that become more prominent in low-quality images. Additionally, some CMR sequences had suboptimal temporal resolution for strain assessment and we suspect that this could have introduced measurement error and reduced statistical power. Future prospective cohort studies with improved spatial and temporal resolution and additional views of the RA would be beneficial to overcoming these limitations. Similarly, incorporation of clinical comorbidities such as DVT/PE, OSA, and COPD, which were not available to us, would be beneficial in future studies. As of the writing of this, use of CMR in patients without clinical CVD is generally limited to research and exploration of the RA with echocardiography could further validate our results for clinical applicability.

## Conclusion

In this large, diverse population free of symptomatic cardiovascular disease at baseline, we found RA maximum and minimum volume indices measured using feature-tracking CMR had an independent positive predictive value for incident AF relative to conventional CVD risk factors and LA parameters. It is unclear if this predictive value persists when ventricular parameters are also taken into consideration. Our findings suggest RA structure among healthy individuals may play a role in the development of AF, and this association should be further investigated.

## Supplementary information

**Additional file 1.** Correlation of AF risk factors and RA volume at study initiation. SD: Standard deviation; BMI: Body mass index. Association of risk factors for AF and RA volume indices is shown. RA volume was generally not significantly different for risk factors though demographic differences by sex and race are evident. Volume is indexed by body-surface area which may affect the significance risk factors due to collinearity.

**Additional file 2.** Association of right atrial volume with incident AF after adjustment for right ventricle variables. EF: emptying fraction; EDM: end-diastolic mass. Models are also adjusted for demographics and traditional risk factors.

**Additional file 3.** Baseline characteristics of patients who did and did not receive cardiac magnetic resonance imaging. Values are mean ± SD or %. CMR: cardiovascular magnetic resonance imaging; SD: Standard deviation; BMI: Body mass index; HDL: High density lipoprotein.

**Additional file 4.** Variation of right atrial parameters at different quality levels, with 95% confidence intervals. Average and confidence intervals shown above for RA parameters at each quality level. RA: right atrium; RAVImax: RA maximum volume index, RAVImin: RA minimum volume index.

**Additional file 5.** Examples of images from sequences excluded from the study for poor quality. Each of these representative images are taken from excluded sequences at RA end-diastole. Each image is from a different study site and was labeled as the long-axis image for that participant. Evident in these images are motion artifact and planes which do not completely capture the RA. Such images were numerous in our analysis, resulting in the exclusion rate.

## Data Availability

The data that support the findings of this study are available from the MESA Coordinating Center but restrictions apply to the availability of these data.

## Abbreviations

AF: Atrial fibrillation
BMI: Body mass index
BP: Blood pressure
BSA: Body surface area
CMR: Cardiovascular magnetic resonance
COPD: Chronic obstructive pulmonary disease
CT: Computed tomography
CVD: Cardiovascular disease
DVT: Deep venous thrombosis
ECG: Electrocardiogram
EF: Emptying fraction
HDL: High density lipoprotein
RA: Right atrium/right atrial
LA: Left atrium/left atrial
LV: Left ventricle/left ventricular
MESA: Multi-Ethnic Study of Atherosclerosis
OSA: Obstructive sleep apnea
PE: Pulmonary embolism
RA: Right atrium/right atrial
RAVI_max_: Right atrial maximum volume indexed
RAVI_min_: Right atrial minimum volume indexed
RV: Right ventricle/right ventricularHR: hazards ratio
SD: Standard deviation
VTE: Venous thromboembolism
