# Supplementary material for: Association of right atrial structure with incident atrial fibrillation: a longitudinal cohort cardiovascular magnetic resonance study from the Multi-Ethnic Study of Atherosclerosis (MESA)
Source: J Cardiovasc Magn Reson. 2020 May 21;22:36. doi: 10.1186/s12968-020-00631-1 (PMC7240918; doi:10.1186/s12968-020-00631-1)
Supplement: Supplementary file 1 — Additional file 1. Correlation of AF risk factors and RA volume at study initiation. SD: Standard deviation; BMI: Body mass index. Association of risk factors for AF and RA volume indices is shown. RA volume was generally not significantly different for risk factors though demographic differences by sex and race are evident. Volume is indexed by body-surface area which may affect the significance risk factors due to collinearity. [file 12968_2020_631_MOESM1_ESM.docx]

Additional file 1. Correlation of AF risk factors and RA volume at study initiation

|  | RA Volume Maximum Index (mL/m^2^) | | RA Volume Minimum Index (mL/m^2^) | |
| --- | --- | --- | --- | --- |
| Risk Factor, continuous | Coefficient | P-value | Coefficient | P-value |
| Age, per year | -0.027 | 0.07 | 0.02 | 0.06 |
| BMI, per kg/m^2^ | -0.02 | 0.44 | -0.06 | 0.007 |
| Risk Factor, categorical | Mean ± SD | P-value | Mean ± SD | P-value |
| Sex Female | 22±7.6 | <0.001 | 22±7.6 | <0.001 |
| Male | 23±8.7 |  | 23±8.7 |  |
| Race Caucasian | 21±7.9 | <0.001 | 11±5.8 | <0.001 |
| Hispanic | 25±8.6 |  | 13±6.4 |  |
| African American | 22±7.4 |  | 12±5.4 |  |
| Chinese | 25±8.5 |  | 14±6.3 |  |
| Smoking status Never | 23±8.2 | 0.82 | 12±5.9 | 0.41 |
| Former | 22±8.0 |  | 12±5.9 |  |
| Current | 21±8.1 |  | 12±6.2 |  |
| Alcohol Use Never | 23±8.4 | 0.11 | 12±5.9 | 0.12 |
| Former | 22±7.8 |  | 112±5.7 |  |
| Current | 23±8.1 |  | 12±6.0 |  |
| Hypertension Medications No | 23±8.2 | 0.87 | 12±6.0 | 0.92 |
| Yes | 22±8.1 |  | 12± |  |
| Diabetes History No | 23±8.1 | 0.91 | 12±6 | 0.23 |
| Impaired fasting glucose | 22±8.1 |  | 13±5.9 |  |
| Diabetic | 22±8.3 |  | 12±5.6 |  |

SD: Standard deviation; BMI: Body mass index

Association of risk factors for AF and RA volume indices is shown. RA volume was generally not significantly different for risk factors though demographic differences by sex and race are evident. Volume is indexed by body-surface area which may affect the significance risk factors due to collinearity.
