# Supplementary material for: Association of right atrial structure with incident atrial fibrillation: a longitudinal cohort cardiovascular magnetic resonance study from the Multi-Ethnic Study of Atherosclerosis (MESA)
Source: J Cardiovasc Magn Reson. 2020 May 21;22:36. doi: 10.1186/s12968-020-00631-1 (PMC7240918; doi:10.1186/s12968-020-00631-1)
Supplement: Supplementary file 2 — Additional file 2. Association of right atrial volume with incident AF after adjustment for right ventricle variables. EF: emptying fraction; EDM: end-diastolic mass. Models are also adjusted for demographics and traditional risk factors. [file 12968_2020_631_MOESM2_ESM.docx]

Additional file 2: Association of right atrial volume with incident AF after adjustment for right ventricle variables.

|  | Adjusting for RV EF | | Adjusting for RV EDM | |
| --- | --- | --- | --- | --- |
| RA Parameter | HR per SD | P-value | HR per SD | P-value |
| RA maximum volume indexed | 1.19 | 0.003 | 1.16 | 0.01 |
| RA minimum volume indexed | 1.12 | 0.047 | 1.10 | 0.09 |

EF: emptying fraction; EDM: end-diastolic mass

Models are also adjusted for demographics and traditional risk factors.
