# Supplementary material for: Association of right atrial structure with incident atrial fibrillation: a longitudinal cohort cardiovascular magnetic resonance study from the Multi-Ethnic Study of Atherosclerosis (MESA)
Source: J Cardiovasc Magn Reson. 2020 May 21;22:36. doi: 10.1186/s12968-020-00631-1 (PMC7240918; doi:10.1186/s12968-020-00631-1)
Supplement: Supplementary file 3 — Additional file 3. Baseline characteristics of patients who did and did not receive cardiac magnetic resonance imaging. Values are mean ± SD or %. CMR: cardiovascular magnetic resonance imaging; SD: Standard deviation; BMI: Body mass index; HDL: High density lipoprotein. [file 12968_2020_631_MOESM3_ESM.docx]

Additional file 3. Baseline characteristics of patients who did and did not receive cardiac magnetic resonance imaging

|  | **Received CMR**  N=5004 (72%) | **No CMR**  N= 1899 (28%) | **P value** |
| --- | --- | --- | --- |
| Age(years), mean ± SD | 64±10 | 62±10 | <0.001 |
| Males, n(%) | 2381(48%) | 832(46%) | 0.23 |
| Race, n(%) |  |  | <0.001 |
| Caucasian | 1957(39%) | 665(37%) |  |
| Hispanic | 1109(22%) | 387(21%) |  |
| African American | 1285(26%) | 608(34%) |  |
| Chinese | 652(13%) | 151(8%) |  |
| Smoker (past or current), n(%) | 2420(48%) | 954(50%) | 0.14 |
| BMI(kg/m^2^), mean ± SD | 27.7±5 | 30±6 | <0.001 |
| Systolic blood pressure(mmHg), mean ± SD | 125±21 | 129±22 | <0.001 |
| Diastolic blood pressure(mmHg), mean ± SD | 72±10 | 72±10 | 0.87 |
| Taking blood pressure medication, n(%) | 1766(35%) | 770(43%) | <0.001 |
| Total cholesterol(mg/dl), mean ± SD | 194±35 | 194±37 | 0.72 |
| HDL(mg/dl), mean ± SD | 51±15 | 50±14 | 0.02 |
| Taking lipid lowering medications, n(%) | 796(16%) | 304(17%) | 0.39 |
| Fasting glucose (mg/dL), mean ± SD | 96±29 | 100±33 | <0.001 |
| Diabetes, n(%) |  |  | <0.001 |
| Impaired fasting glucose | 645(13%) | 294(16%) |  |
| Diabetes | 581(12%) | 278(15%) |  |
| Alcohol Use |  |  | 0.03 |
| Current | 2795 (55%) | 954 (53%) |  |
| Former | 1154 (23%) | 470 (26%) |  |

Values are mean ± SD or %.

CMR: cardiovascular magnetic resonance imaging; SD: Standard deviation; BMI: Body mass index; HDL: High density lipoprotein
