# Supplementary figures and images for: Association of right atrial structure with incident atrial fibrillation: a longitudinal cohort cardiovascular magnetic resonance study from the Multi-Ethnic Study of Atherosclerosis (MESA)
Source: J Cardiovasc Magn Reson. 2020 May 21;22:36. doi: 10.1186/s12968-020-00631-1 (PMC7240918; doi:10.1186/s12968-020-00631-1)

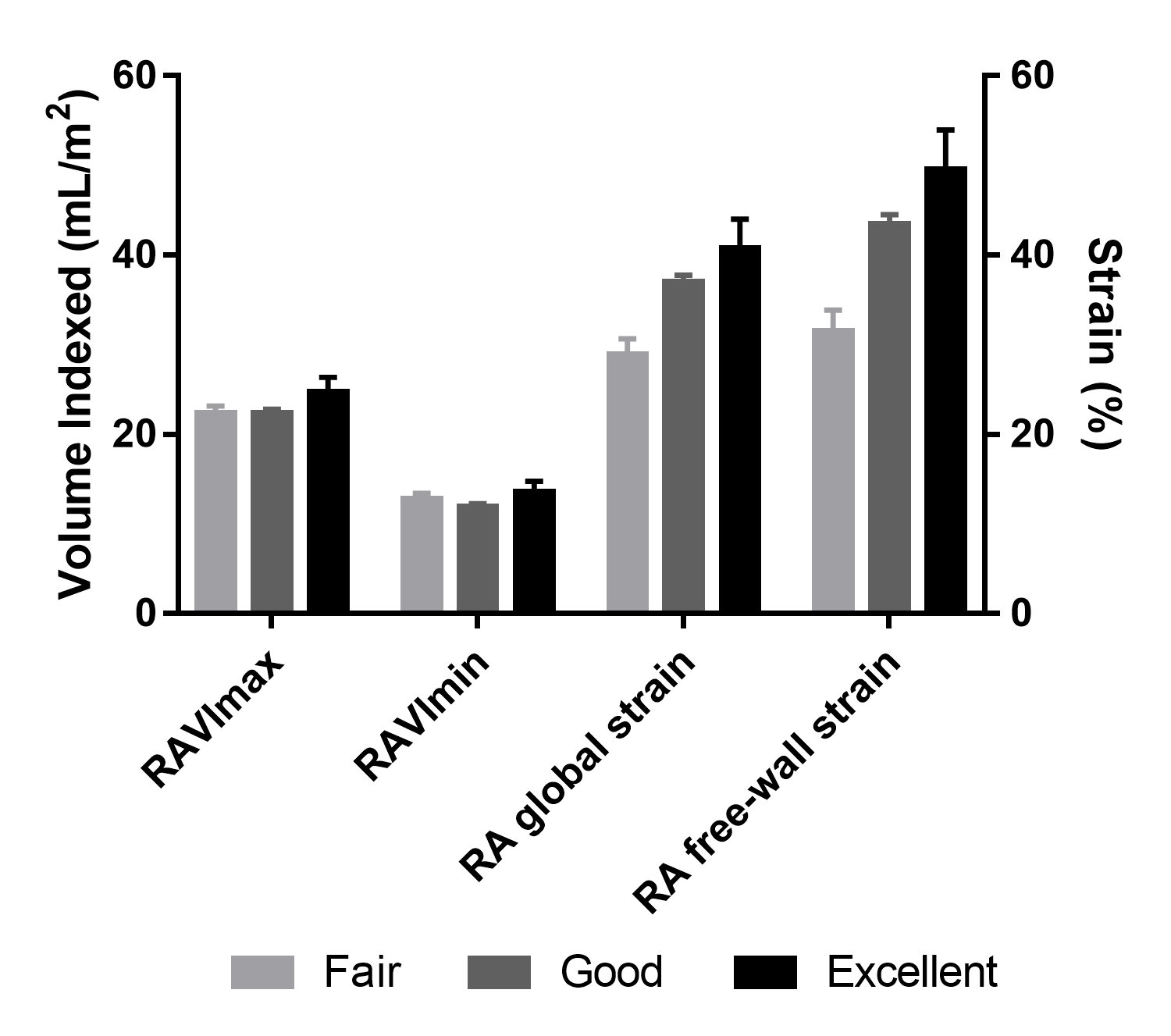

Supplement: Supplementary file 4 — Additional file 4. Variation of right atrial parameters at different quality levels, with 95% confidence intervals. Average and confidence intervals shown above for RA parameters at each quality level. RA: right atrium; RAVImax: RA maximum volume index, RAVImin: RA minimum volume index. [file 12968_2020_631_MOESM4_ESM.tif]
